# Maternal and newborn health for the urban poor: the need for a new mental model and implementation strategies to accelerate progress

**DOI:** 10.1186/s12992-022-00830-8

**Published:** 2022-04-28

**Authors:** Shanon McNab, Elaine Scudder, Uzma Syed, Lynn P. Freedman

**Affiliations:** 1 Averting Maternal Death and Disability Program, Columbia University Mailman School of Public Health, New York, NY USA; 2 Save the Children US, Washington, DC USA; 3 Save the Children International, Washington, DC USA

**Keywords:** Urbanization, Maternal and newborn health, Implementation, Slum

## Abstract

**Background:**

Urbanization challenges the assumptions that have traditionally influenced maternal and newborn health (MNH) programs. This landscaping outlines how current mental models for MNH programs have fallen short for urban slum populations and identifies implications for the global community. We employed a three-pronged approach, including a literature review, key informant interviews with global- and national-level experts, and a case study in Bangladesh.

**Main body:**

Our findings highlight that the current mental model for MNH is inadequate to address the needs of the urban poor. Implementation challenges have arisen from using traditional methods that are not well adapted to traits inherent in slum settings. A re-thinking of implementation strategies will also need to consider a paucity of available routine data, lack of formal coordination between stakeholders and providers, and challenging municipal government structures. Innovative approaches, including with communications, outreach, and technology, will be necessary to move beyond traditional rural-centric approaches to MNH. As populations continue to urbanize, common slum dynamics will challenge conventional strategies for health service delivery. In addition, the COVID-19 pandemic has exposed weaknesses in a system that requires intersectoral collaborations to deliver quality care.

**Conclusion:**

Programs will need to be iterative and adaptive, reflective of sociodemographic features. Integrating the social determinants of health into evaluations, using participatory human-centered design processes, and innovative public-private partnerships may prove beneficial in slum settings. But a willingness to rethink the roles of all actors within the delivery system overall may be needed most.

## Introduction

A new reality is shaping maternal and newborn health (MNH) in the twenty-first century: the world is rapidly urbanizing, which requires a fundamental shift in the mindset that structures MNH strategies. This is especially true in light of the push for Universal Health Coverage (UHC), and amid the COVID-19 pandemic, which have had disproportionate impacts on access to health services for people living in slums [1, 2]. It entails questioning longstanding assumptions that undergird rural health programs, including assumptions about social structures, leadership structures, what women want and how they access information and services, and how change happens – or is resisted. Our thinking about urban MNH should also reflect broader trends affecting global health and development, including the shift from a primary focus on effective technical interventions to an agenda that also centrally includes implementation challenges. Specifically, urban MNH strategies should recognize the need to engage with the broader context of people’s lives and to shift the power to frame and solve problems from the exclusive hold of technical experts and centralized political leaders to the people and providers whose interactions define the ultimate impact of health inventions on women and newborns. As the development community continues to attempt to focus on decolonization, localization and genuinely shifting power to women and providers who are directly impacted, the urban MNH should be a part of this movement [3].

These very real implementation challenges and failures to reach the most vulnerable have begun to surface in recent years. The 2015 State of the World’s Mothers Report found that where child survival gaps are largest, poor urban children are 3-5 times as likely to die as their affluent peers [4]. Globally, while 98% of stillbirths occur in low- and middle-income settings, 40% occur in urban settings [5]. In Kenya, the maternal mortality ratio (MMR) of 706  per 100,000 live births in Nairobi slums dwarfs the national average of 362 per 100,000 live births. Women living in slums in Bangladesh are more likely to experience pregnancy-related complications than women who do not live in slums [6]. In Kenya, Ecuador, Brazil, Haiti, and the Philippines, children in slums are more likely to die during the first year of life than those in rural areas [7, 8].

Despite the clear necessity to better deliver services and meet the needs in these contexts, which has led to increased attention to urban health and poverty, data on MNH in slums remains sparse [9]. There may be an assumption that insights related to urban settings will also be applicable to slums, but given the unique environment, social circumstances, and health considerations in slums, data on urban areas more generally are  not likely to be representative of the situation in slums [10, 11]. This literature imbalance may begin to shift, as the COVID-19 pandemic has not only prompted new thinking on routine MNH care seeking and service delivery, but also on how slum residents may be disproportionately affected by lockdowns and infection prevention efforts, such as social distancing [2, 12]. However, there remains a paucity of literature at the intersection of maternal and urban health [9]. What data do exist show a need to examine slum health independent of urban health and to support MNH programming explicitly designed for slum dwellers [6, 13].

These harsh realities highlight that the programmatic assumptions and intervention strategies that are predominantly being used by implementing MNH partners – herein referred to as the current ‘mental model’ – are clearly not working in these evolving contexts.

### What is the current ‘mental model’?

The global ‘mental model’ for providing effective MNH services is often the foundation for most urban programming. The model is generally based on the following premises: 1) there is a defined catchment area: it is clear who the community is and who the thought leaders are; 2) trusted social networks exist and can be identified by most of the community leaders; 3) the role of the government is relatively clear and their responsibility to provide care is understood; 4) the public healthcare-seeking pathway from community to tertiary care is understood (though not necessarily followed); 5) ascertaining implementing partners rolels is primarily done through existing coordination platforms; and, 6) paid employment opportunities are fewer: a model based on community health volunteerism has worked.

Addressing these increasing gaps in urban health will require open minds, better collaboration, and critical thinking from all of us. With this challenge in mind, Save the Children (SC) partnered with the Averting Maternal Death and Disability (AMDD) program at Columbia University with the aim of conducting a global landscaping of the state of MNH programs in urban slums in 2015-2016.

## Methods

This situation analysis employed a three-pronged approach, consisting of a literature review, key informant interviews, and a case study in Bangladesh.

Literature reviews were conducted using PubMed, Google, and Google Scholar to identify basic urban MNH background information, recent urban research conducted, program evaluations, policy papers, and other relevant contextual information. Websites of large MNH programs, donors, and urban-focused initiatives were also searched. A critical interpretive synthesis (CIS) of the literature was employed [14]. The CIS methodology allows for ‘insights and interpretations drawn from a broad range of relevant sources’ and is well suited for topics for which literature is not as well developed [15], such as urban MNH. This granted a broader understanding of urbanization, informality, modernity and other themes that were important for understanding the context [15–18]. From this understanding, a conceptual framework was developed to further guide analysis of the research findings, and to identify implications for a way forward for policy and programming for MNH in urban settings.

A systematic review was then conducted to identify prominent MNH programs targeting urban poor. The aim of the review was threefold: 1) to identify facilitators and barriers to effective implementation; 2) to generate evidence to refine the conceptual framework; and 3) to assess the current strengths and gaps of the existing research base. An extraction tool was completed for each of the programs identified, capturing 12 categories.^1^ Formal program evaluations were then reviewed, and a narrative synthesis was conducted to gather data on the effectiveness of these programs [19]. The synthesis identified 1) successful components or facilitating factors; 2) programmatic failures and reasons identified; and 3) challenges encountered in the implementation. To better understand how key urban-relevant policies shape the context for MNH programs, a purposive search for policies was conducted in select countries, and a documentary analysis was employed to assess the role that specific policies and policy gaps play in the implementation and effectiveness of urban MNH programs. This approach allowed us to deepen the understanding of what works in the urban context and why.

Throughout these stages, key program staff and relevant stakeholders were identified. Key informant interview (KII) guides were then drafted and KIIs were undertaken with various informants: SC headquarters and country staff, global MNH experts, urban experts, and donors. In total, 22 KIIs were conducted as part of the global scoping, and an additional 14 KIIs were conducted in Bangladesh as part of the case study. That case study report is available separately, although some of its findings are referenced here.

**Table:** 

**Challenges with Terminology** Throughout this review, we compiled a glossary of common terms, including the following: urban, slum, urban population, urban poor, city, town, slum-like settlement, slum household, informal settlement, informal sector, informal work, informality, and squatter settlement. It became apparent that there is no global consensus on the definitions of key terms; often the key terms – such as “urban” or “poor” – are not defined at all. This makes most comparisons across cities, countries, and trends over time impossible to state with certainty. In fact, it makes most of the quantitative data in the literature unreliable [9, 20]. For that reason, no paper or other evidence was excluded from consideration because it failed to meet a particular definition of “slum” or “urban poor.” In the text, we use “slum” and “informal settlement” interchangeably and vacillate between “slum” and “urban poor” depending on whether we are talking about the place or the people. This is done to reflect the way that these terms are usually used by the literature and by our informants.	

## Results

Below we present our findings on the inadequacies of the current mental model in meeting the needs of the urban poor.

### How the current mental model falls short with the urban poor

#### The urban slum population is heterogeneous and highly mobile

Within any given slum, residents may differ on economic status, statehood or citizenship, language, religion, ethnicity, or length of residence (Participant 1, personal communication, January 23, 2016; Participant 2, personal communication, January 12, 2016) [21].This diversity limits the extent to which a ‘typical slum dweller’ can be described; there is “not one model, not one condition, not one way to think of the urban poor,” (Participant 3, personal communication, March 7, 2016). Key informants spoke of the need to respect and understand the heterogeneity of slum residents, particularly women, and how these differences would impact health care access and seeking, as well as health outcomes.

In addition, the amount of diversity and social interaction within slums often varies. For example, residence patterns in Lagos slums are not dictated by tribe or language, leading to a diffuse environment and fewer cultural ties between residents (Participant 2, personal communication, January 12, 2016). While the benefits of diversity in urban areas are often heralded, key informants also stated that diverse settings can lead to weaker social networks and disconnect between slum residents (Participant 3, personal communication, March 7, 2016) [22].

The highly mobile nature of urban populations also impacts social cohesion and program implementation. BRAC staff associated with the Manoshi program in Bangladeshi slums estimated that, within one year, 20-40% of the slum population had moved [23]. Furthermore, both men and women often leave the slum for many hours of the day for work or errands, further constraining programs’ access to slum residents (Participant 4, personal communication, March 2, 2016). Respondents noted that this mobility made defining and locating community members for ‘community-based’ programs extremely challenging, unless very narrowly defined and small in scale.

#### Urban power structures are complex and ever changing

The sociodemographic dynamics in slums are arguably more complex and rapidly changing than those in more stable rural areas. “Strong epidemiological evidence suggests that individuals with diversified social networks who interact with family members, friends, neighbors and fellow workers, are married, or belong to social and religious groups, live longer and healthier lives than those who are less socially embedded and involved” [22].

##### Social networks

Increasingly, work has been done to show the value of ‘diverse social networks’ for women [24]. The positive outcomes associated with strong social networks include increased likelihood of delivering with a skilled birth attendant and attending postnatal care sessions [24]. The literature on social support systems in slums most relevant to MNH is that  focused on the role of friendship between mothers and peer educators, health workers, and/or community health workers. In Mumbai slums, social capital has been identified as crucial during health crises, allowing women to navigate complex networks of public and private clinics [25].

Despite this positive association, much of the literature shows that women in slums often have weak and unreliable networks, described as fragile, non-existent, and incubators of misconceptions and poor practices – especially around newborn health and family planning [26–29]. Key informants confirmed that women in urban slums are vulnerable in ways that they are not when surrounded by family and community in rural areas, including being more easily victimized, and without a strong sense of support (Participant 2, personal communication, January 12, 2016; Participant 4, personal communication, March 2, 2016). That said, moving from slums into public housing as part of a slum rehabilitation program has been found to lessen women’s social ties further, suggesting community and social capital in slums may be disrupted by rehousing efforts [30].

##### Leadership

Slums and slum-like settlements are often built without formal approval and are considered illegal, which often complicates the ability of residents to claim access to public services, including health care. Furthermore, slum health often falls under the authority of the municipal government, and our KIIs emphasized that municipalities are often not equipped (nor prepared) to serve the diverse needs of the growing population. In place of this, leadership structures common in slums can grant informal actors tremendous power and present unique challenges. In addition, the lack of clear legitimate structures makes it hard for outside organizations to navigate the slums. In the Bangladesh case study, we found that residents must contend with exploitative *mastaans* (“musclemen” or thugs) to access water, sanitation, electricity, and health services, and international non-governmental organization (INGO) representatives spoke of the importance of working with power brokers to implement programs. Other interviewees described challenges with the lack of clarity around who is ‘in charge’ and thus the need to spend more time earning the trust of women and communities. As seen in other contexts, the time needed to identify key stakeholders and get their buy-in can be significantly longer than in rural areas [31]. Still, informants agreed that taking the time to identify sources of power and influence was fundamental to any work in slums (Participant 2, personal communication, January 12, 2016; Participant 4, personal communication, March 2, 2016).

Power structures within slums are difficult to navigate. Few residents have the option to organize and advocate for change, demand services, or engage with the local and national level government [31, 32]. Informal and fluid leadership takes the place of more traditional hierarchies; power dynamics between slum dwellers can dictate issues of access, information, and inclusion; and often the residents are economically and politically isolated from the larger city. Even more challenging is the limited sense of community from within [32]. Researchers argue that “slum dwellers are unable to demand services owing to weak community organization and low collective confidence that is known to increase utilization of health services.” [33] This purported ‘weaker’ sense of community has prevented many slum dwellers from demanding higher quality services, improved infrastructure, and inclusion.

**Table:** 

**Women’s Groups: do they translate in the urban slums?** Various trials have tested the effectiveness of women’s groups to improve MNH [34]. From 2006 to 2009, the City Initiative for Newborn Health (CINH) was a cluster randomized controlled trial in Mumbai slums and was one of several trials testing “women’s groups” for reducing newborn mortality [34]. In this trial, women’s groups were established to engage in a participatory learning and action process, to discuss perinatal health concerns, and to take group action. Unlike the Ekjut trial of women’s groups in rural areas of Jharkhand and Odisha (formerly Orissa), India, which demonstrated a substantial reduction in newborn mortality [35], the CINH trial found the intervention had no impact on newborn mortality [22]. The researchers speculated that this may be due to social conditions within the slum or larger sociopolitical issues. While women were initially enthusiastic about participating, they “were less successful in undertaking collective action such as negotiations with civic authorities for more amenities.” Among the most important findings was that “[g]roup members helped others individually but balked at collective strategizing” [22]. This has important implications for interventions looking to improve newborn health and wellbeing in urban settings. Outside of health, there is a growing literature on the ways in which people living in informal circumstances strategize individually to skirt the law in order to survive; for example, illegally tapping electric lines for power, or selling snacks on the street. Whereas community health interventions in rural areas often build upon social networks in which community ties support individual health, the same cannot be assumed^2^ to operate in urban areas, and particularly in slums. A major limitation of the literature reporting that women’s groups effectively reduce maternal and neonatal mortality is that they were designed “primarily on a rural development framework,” and there is insufficient evidence to suggest that this model works in the urban context [37].	

#### The public health sector is nonexistent or of poor quality in urban settings

We did not find any examples of a functioning public health system with comprehensive services tailored to or easily accessible by slum dwellers; in our interviews, the public sector was often described as ‘nonexistent’, of poor quality, inconsistently available, or only used in emergencies. Although public tertiary hospitals typically exist in big cities, there is often no clear, linear hierarchy of lower-level clinics and dispensaries to serve the urban areas. The senior health representative of City Corporation Dhaka South explained that the public sector in Dhaka is dramatically understaffed, overcrowded, and overburdened, and tertiary hospitals are overwhelmed by self-referrals (Participant 5, personal communication, December 9, 2016). And another informant noted ‘though there are facilities, there is no access” (Participant 9, personal communication, December 26, 2016). Though not representative of all public sectors, these findings resonated with many of the implementing partners. Lack of referral systems reportedly made it nearly impossible for implementers to identify a platform for delivering MNH interventions in a way that ensures household-to-hospital continuum of care.

This limited public sector service delivery in slums leaves a vacuum that has been filled by the private sector. Research shows, and respondents confirmed, that many slum dwellers are increasingly seeking care from the private sector, including unregistered healthcare providers, traditional healers, “quacks”, and drug sellers [38]. As private sector care is usually more expensive, slum populations are reportedly spending an increasing percentage of their incomes on out-of-pocket medical costs (Participant 6, personal communication, January 25, 2016) [1, 39]. One study in Gujarat, India found that a majority of women residing in slums paid up to three times more money and 20% of family income to give birth in a private hospital as opposed to a government hospital [40]. And, these providers often enter slum markets without the necessary expertise to tackle the challenges at hand (Participant 3, personal communication, March 7, 2016). They often operate outside of the formal regulatory structure and with little accountability. Studies of private sector facilities in urban areas have found the staff to be unqualified or untrained; function without supervision; lack equipment, drugs, and infrastructure; and ultimately unable to provide basic life-saving services for mothers and newborns [41–43]. Despite this, unlicensed providers are typically slum dwellers' ‘first point of contact with the health system’ [44–46].

Hence, a commonly cited challenge was figuring out how to acknowledge the presence of the private sector while continuing to improve the quality and availability of the public sector. The overwhelming sentiment among key informants, and aligning with the Lancet series on UHC, was that the private sector must be engaged if health outcomes in slums are to improve (Participant 7, personal communication, March 10, 2016; Participant 4, personal communication, March 2, 2016) [47].

#### Women’s accessibility is limited and service provision needs to be more agile

As in rural areas, women in slums often work long hours; however, this work is often outside of the slum and the opportunity cost of missing work to seek care may have more immediate negative implications. One informant described how in Nairobi, high unemployment rates undercut job security: if a mother is working as a cleaner, a job with a high replacement rate, she cannot miss work to take herself or her child to the doctor without risking unemployment (Participant 4, personal communication, March 2, 2016) [48]. Often, immunizations or follow-up visits are sacrificed by women to maintain employment [46, 48]. In Ethiopia, the Urban Health Extension Workers have voiced frustration that they cannot find women in their homes during the day, making their roles more challenging and less fulfilling (Participant 6, personal communication, January 25, 2016). This sentiment was shared by informants in Bangladesh, Kenya, and India.

**Table:** 

**No easy solution to reaching women** INGOs implementing MNH programs in urban slums shared some of the strategies they tested, which often failed, to adequately adjust to reflect women’s availability. One solution that was tried by Marie Stopes and the Urban Primary Health Care Services Development Project (UPHCSDP) Clinics in Bangladesh was extending the hours of the clinics. However, due to additional bottlenecks, this led to varying success: some women felt unsafe walking at night, so the hours had no effect on their care-seeking (Participant 8, personal communication, December 6, 2016; Participant 4, personal communication, March 2, 2016), while some clinicians objected to staying late, especially when the facilities were located in the slums (Participant 8, personal communication, December 6, 2016; Participant 6, personal communication, March 2, 2016).	

## Discussion

Our observations are meant to help shift the way in which MNH is addressed in urban slums, where the primary learning needs to be about how the dynamics of the urban context shape the ecology of implementation. This calls for shifts in the way that global and national level policies, program designs, and implementation strategies aim to understand and improve health outcomes for the urban poor.

### Implementation challenges that remain

The implementation challenges that will have to be addressed in earnest can be categorized into two groups: the hardware and the software [49].

#### Hardware

The lack of quality, disaggregated data to inform policy, program, and practice impacts the ability to target or even identify the most vulnerable and prevents strong program/research design [50]. The ever-changing sociodemographic composition of slum populations poses several challenges to standard program implementation, requiring a flexible, adaptive, and iterative approach. Slum populations may consist of multiple or competing ethnicities, languages, cultural norms, and religions. Coordination amongst implementing NGOs, government, and private organizations within the health sector and across sectors– education, infrastructure, employment, law enforcement, the environment – is largely missing. The need to work with municipal governments who have limited capacity and resources for delivering health services targeting the urban poor has prevented progress in many slum settings.

#### Software

There is a fundamental issue of trust; slum dwellers’ inherent distrust of ‘outsiders’ was a common theme. The need to have links and relationships with a community that is quite transitory remains a challenge, but programs are more likely to fail without this relationship. Social networks were differently organized and difficult to tap into, challenging interventions that rely on bringing women together often and via traditional leadership. Given the heterogeneity of the communities, there is no one successful communication or outreach method. In contrast to rural areas where radio campaigns and village events are often used, behavior change messaging campaigns are complicated in urban areas using myriad communications channels and technologies, as well as competing and often contradictory messages.

### Implications for moving forward

Using a program design framework below, we present questions for key stakeholders to answer with thoughtful considerations about the unique challenges of meeting the needs of the urban poor, particularly in slum settings.

#### What intervention and implementation strategies?

Programs for urban slums need to employ a participatory, human-centered design process that puts high value on slum dwellers’ perspectives. Fortunately, the current nascent nature of the urban MNH space makes it fertile ground for such design. Alongside policy-level changes, programs should build up from the realities of slum settings rather than a top-down effort to implement so-called evidence-based interventions. This is an important lesson from past efforts to take RMNCAH programs developed in and designed for rural settings and import them wholesale into urban settings [31]. To be effective, MNH programs in urban slums will need to address different social dynamics, individual aspirations, environmental and physical constraints, resource availability, and financial pressures. That said, it is equally important to recognize that no perfect program encompassing all aspects of slum development exists; promising solutions to improve slum health should not be delayed while searching for the ‘ideal’ initiative [13].

#### For what population?

To improve equitable coverage, implementers must consider creative ways to enumerate the households and populations in the catchment area and track cases. The challenges of doing this for highly mobile populations living in illegal housing in unofficial settlements and engaging in informal employment are daunting. Mobile and other digital methods hold promise. One example is the Toolkit for Health Urban Life in Slums Initiative that is using a mobile application to track health conditions at the household, family, and individual level in Bangalore slums [51]. A systematic review of census methods for temporary populations found that mobile phone interventions are the fastest growing medium for enumerating populations; however, census and survey data remain “an important backbone” through which temporary population estimates are derived [52]. Mobile phone interventions for children’s immunizations in slums have been piloted in Guatemala and Bangladesh and preliminary results suggest that these are both feasible and effective in improving vaccination rates among those who have been otherwise difficult to locate given their mobility [53, 54]. Other information and communications technology (ICT) approaches mediums may be effective for specific health initiatives; future research should seek to understand the potential of more advanced mHealth applications beyond SMS for urban populations [54]. While the field remains open for creative new ideas, and COVID-19 is accelerating the need and use of ICT, issues of privacy, phone ownership, and women’s access to phones should be considered [53–55].

#### Delivered through which providers?

Ultimately, a key issue is whether an accepted or successful service delivery platform for MNH interventions exists, requiring an understanding of how and why the urban poor access services. The Bangladesh case study presented some extremes, but the notion of slum health being a ‘pariah’ and ‘unwanted problem’ came up in many interviews, indicating that the complex and inter-connected nature of slum health is not a problem that governments want or are equipped to handle. With the absence of a robust public health system this will inevitably force implementers to confront the extensive reach in urban slums of small-scale, unregulated, for-profit private providers. A fundamental choice for implementers is whether to accept the reality of the private sector’s dominance and attempt to work with them, or instead to develop an alternative network of providers in the hopes of drawing the urban poor to it.

#### With what outcomes?

Beyond standard MNH outcomes related to health status or intervention coverage, it might be important to better understand how to address dynamics of care-seeking and service utilization. Our scoping highlighted deep disconnects between users (slum communities) and the health system, such as low rates of intervention coverage, lack of knowledge, distrust, expectations of poor-quality treatment, and fear of catastrophic health costs. Mental health is also worth capturing, as research suggests high rates of post-partum depression, intimate partner violence, and stress related to delivery costs and other factors in slum populations [56, 57]. Approaches that integrate health outcomes and social determinants of health into evaluations – such as Health Impact Assessment (HIA) and Health in All Policies (HiAP) – may be beneficial [58].

#### Measured how?

The metrics and measurement strategies used depend on the intervention and implementation strategies being employed. This may be an area where presence in densely populated cities, with good internet and cellphone coverage, might open the possibility for creative new uses of digital health technologies to improve data collection and tracking [51]. In areas where cell phones are not owned or controlled by women, or where residency is even more tenuous, creative use of local groups and mobile units may be tested.

#### With what potential for scaling and sustainability?

The need to scale in a sustainable manner will no doubt pose unique challenges for urban slum health. The mobility of populations makes any community-based organizing inherently difficult. The deep insecurity of tenure when housing is informal/illegal is likely to have profound effects on the willingness of people to invest their time, energy, and trust in health service programs. And the fact that generally weak municipal governments have formal responsibility for urban health compounds the challenge. In nearly all interviews, there was a clear sentiment that working with a municipal government is ‘fundamentally different than working with rural governments’ (Participant 10, personal interview, February 19, 2016).

## Conclusion

Given these real challenges, we question if there is a need to start over or if the current model can flex appropriately. Urban dynamics will challenge many of the conventional strategies for MNH, as the epicenter of the sector will increasingly need to move to cities, where poor and marginalized people live in circumstances that bear little resemblance to the stable, rural villages where existing MNH practice has taken shape. Many respondents noted that proposed ways forward for delivering services – harnessing technologies, working with non-traditional partners, and adjusting implementation strategies - would be innovative but would also challenge standard approaches. Several expressed concern about the inability, especially of development partners, to think and work outside of their comfort zones – particularly within the context of the informal sector.

The COVID-19 pandemic has further exposed cracks in the global MNH system, especially when considering the loss of progress faced in almost every country, shining a light on the need for a nimble and localized system. This will continue to be true — perhaps even more so — in informal urban and peri-urban settlements. The urban poor are particularly vulnerable given the reliance on the informal sector for livelihoods which has been decimated in many places; their proximity to one another preventing feasible social distancing; and the fears of transmission have kept an already weary population outside of the health system when they may need it most [59, 60].

The need to be responsive to this changing reality, to think outside of traditional public health strategies, and engage with populations that are hard to find, quantify, and reach, will prove a mighty challenge. Yet in the face of this new reality, the need to act on commitments to UHC with quality and equity, to build inclusive and sustainable cities, and to advance human rights-based approaches to development remains relevant and pressing. If the world is to be true to these commitments, the door must swing open for creative new approaches to achieving good health for the urban poor, and we should start with the MNH community.

## Data Availability

The datasets generated during the current study are available from the corresponding author on reasonable request.

## Abbreviations

AMDD: Averting Maternal Death and Disability
BRAC: Bangladesh Rural Advancement Committee
CINH: City Initiative for Newborn Health
COVID-19: Coronavirus disease 2019
CIS: Critical interpretive synthesis
HIA: Health Impact Assessment
HiAP: Health in All Policies
RMNCAH: Reproductive, maternal, newborn, child, and adolescent health
ICT: Information communications technology
INGO: International non-governmental organization
KIIs: Key informant interviews
LMICs: Low- and middle-income countries
MMR: Maternal mortality ratio
MNH: Maternal newborn health
NGOs: Non-governmental organizations
SC: Save the Children
UHC: Universal health coverage
UPHCSDP: Urban Primary Health Care Services Delivery Project

## Footnotes

1 The 12 categories included in the extraction tool are as follows: program title, country, donor, duration, relationship with government, target population, implementing partners, activities, impact assessments, other evaluations conducted, linkages with other sectors, and program funding.

2 We recognize that in some settings, women’s groups in slums did empower marginalized groups; however, this result should not be assumed in the slum context [36].
